# Comparison of Postoperative Recovery after Manual and Target-Controlled Infusion of Remifentanil in Bariatric Surgery

**DOI:** 10.3390/medicina57101114

**Published:** 2021-10-16

**Authors:** Greta Kasputytė, Paulina Gecevičienė, Aurika Karbonskienė, Andrius Macas, Almantas Maleckas

**Affiliations:** 1 Department of Anaesthesiology, Lithuanian University of Health Sciences, 50161 Kaunas, Lithuania; paulinaaldak@gmail.com (P.G.); aurika.karbonskiene@kaunoklinikos.lt (A.K.); andrius.macas@kaunoklinikos.lt (A.M.); 2 Department of Surgery, Lithuanian University of Health Sciences, 50161 Kaunas, Lithuania; almantas_maleckas@yahoo.com

**Keywords:** remifentanil, bariatric surgery, postoperative recovery

## Abstract

*Background and Objectives*: Early postoperative recovery after surgery is a key point for patients’ safety and comfort. Moreover, operating room turnover depends on recovery time. Our aim was to assess which method of remifentanil administration, manual (MI) or target-controlled infusion (TCI), could reduce patient time in recovery room. In this study, patients’ recovery times were registered and compared among the groups. *Materials and Methods*: We enrolled 31 morbidly obese patients in this prospective study. All of them had undergone bariatric surgery at the Hospital of Lithuanian University of Health Sciences Kauno Klinikos in 2020. Sevoflurane/remifentanil anaesthesia was performed for all patients. The patients were randomly assigned to the manual infusion (MI) (control group) or target-controlled infusion (TCI) group for the method of the administration of remifentanil. While the patients were waking up after the surgery, we recorded spontaneous breathing and airway reflexes recovery time, time of extubation, eye opening, recovery of orientation and beginning of the following oral command. For the TCI group, we also documented remifentanil concentrations in the blood (automatic infusion pump). *Results*: Patients did not differ in demographic values and duration of remifentanil infusion. We found that remifentanil consumption in the TCI group was lower, *p* = 0.02. Despite lower remifentanil consumption in the TCI group patients, they demonstrated longer total recovery time than the control group patiens: 14 (12–20) vs. 10 (6–16), *p* = 0.001. *Conclusions*: The study showed that, upon comparing the TCI method with MI, manual infusion produced better results in postoperative patient recovery. Additionally, higher doses of remifentanil were consumed using MI. In conclusion, the dosage recommended by highly qualified anaesthesiologists is favourable for morbidly obesity patients when compared to the TCI method.

## 1. Introduction

Morbid obesity is one of the most pressing problems of the 21st century, with the number of obese patients growing rapidly around the world and doctors of different specialisations increasingly encountering patients with morbid obesity in their clinical practice [1]. In 1998, the U.S. National Institutes of Health recommended bariatric surgery as a first-line treatment for morbidly obese patients, and such surgeries have increasingly been performed since then [2]. In the operating theatre, the anaesthesiologist has the particularly complicated task of determining the safest dose of medication and ensuring the most effective perioperative analgesia, sedation, relaxation and a seamless postoperative course. Usually, anaesthetic medicines are dispensed according to the patient’s body weight, but this rule does not apply to morbidly obese patients. Establishing a relationship between the clearance and distribution volume of drugs and the anthropometric data of the individual is a major pharmacokinetic challenge when administering anaesthetics to morbidly obese individuals [3]. Experience has shown that the circulatory and respiratory conditions and metabolism of obese patients, even those with the same BMI, can vary significantly, and additional criteria should be sought to select the appropriate dose. The main objective of this study was to assess which method of remifentanil administration, manual infusion (MI) or target-controlled infusion (TCI), could reduce patient time in the recovery room. 

## 2. Materials and Methods

Held in 2020, the prospective study was hosted by the Hospital of the Lithuanian University of Health Sciences Kauno Klinikos. After having obtained the written consent of the patients and the approval of the local Ethics Committee (BEC—MF—39), 31 morbidly obese patients were enrolled in the study. The inclusion criteria were: II or III ASA physical status, >18 years, gastric bypass surgery with general remifentanil anaesthesia. Study exclusion criteria were: a history of opioid usage to treat chronic pain, opioid dependence, and age under 18 years. 

The manual infusion (MI) (control group) or target-controlled infusion (TCI group) methods were randomly assigned to the participants for the administration of remifentanil. The sealed envelope method was used for the randomisation of the subjects. 

After establishing standard anaesthesia monitoring, including pulse oximetry, electrocardiogram, non-invasive blood pressure and capnography, and obtaining peripheral venous access, preoxygenation was performed. Then, anaesthesia was induced with intravenous (i.v.) fentanyl 1.2–1.5 mcg/kg, i.v. propofol 2 mg/kg and i.v. cisatracurium 0.1 mg/kg. Following tracheal intubation anaesthesia was maintained with sevoflurane (0.7–0.8 MAC) in all patients.

Patients of the MI group received remifentanil infusion 0.2–0.5 mcg/kg/min and of TCI group 4–6 ng/mL using the Minto model (plasma mode, Perfusor^®^ Space, BBraun, Germany). Additionally, to maintain hemodynamic stability based of the expert opinion of the experienced anaesthesiologist in charge of the patient (based on individual patient’s response), norepinephrine 0.01–0.04 mcg/kg/min was infused.

A mixture of 60% of oxygen and air in pressure control mode was used to ventilate the lungs, thereby maintaining normocapnia (EtCO_2_ 35–45 mmHg).

A total infusion of 500 mL Ringer’s Lactate and 500 mL 10% Dextran solutions were administered to all patients during general anaesthesia.

Postoperative analgesics were administered in the operating room: a 0.25% bupivacaine infiltration into the laparoscopic trocar insertion site, i.v. acetaminophen 1000 mg and i.v. ketoprofen 100 mg before the end of surgery. I.v. morphine 5 mg was administered at the end as an alternative opioid of remifentanil infusions.

During early recovery, we evaluated patients’ haemodynamics (arterial blood pressure (BP), heart rate, saturation), recovery time of spontaneous breathing and airway reflexes, time to eye opening, extubation, recovery of orientation and following of verbal commands.

For the TCI group, we also documented calculated remifentanil concentrations in the blood (according to automatic infusion pump). 

We used Statistical Package for Social Sciences (SPSS) software version 23.0 to conduct statistical analyses. Continuous variables were presented as median ± min/max values and categorical variables were summarised as frequencies. The Mann–Whitney U test was used to evaluate continuous variable differences between the groups, and the Pearson χ^2^ test was used to compare categorical variables. A *p*-value less than 0.05 was considered significant. 

## 3. Results

**Figure 1:**
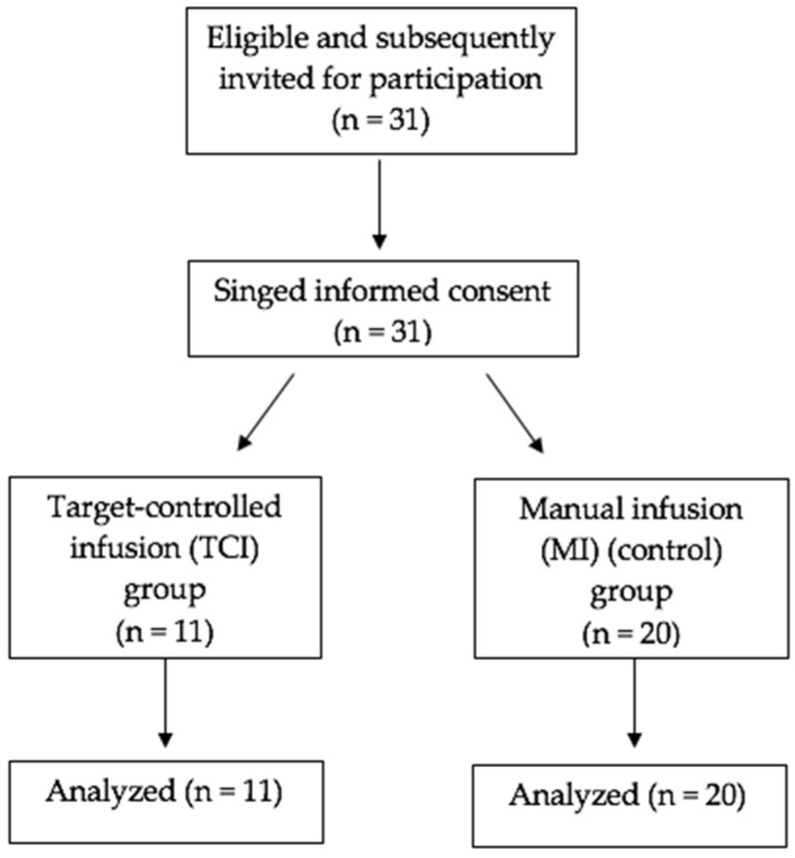
Flow chart for patient enrolment.

Thirty-one patients were enrolled in the study (see Figure 1). 

Patients’ demographic data are shown in Table 1. There were no significant differences in sex, age, body mass index and lean body mass between the two groups. 

Duration of remifentanil infusion during the anaesthesia are shown in Table 2. 

**Figure 2:**
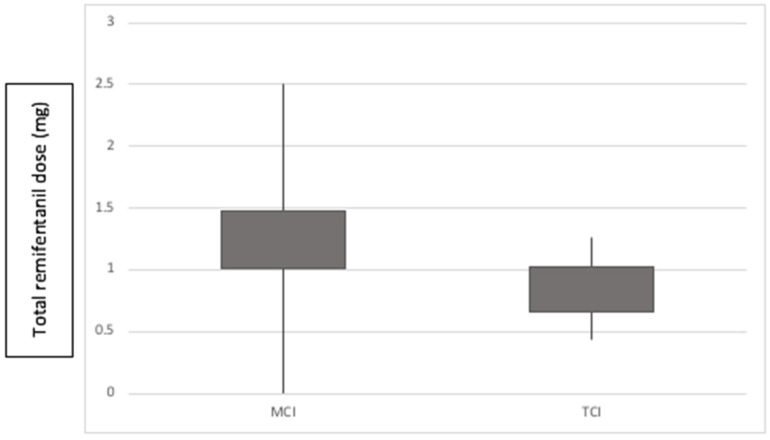
Remifentanil consumption, *p* = 0.02.

We found that consumption of remifentanil (mg) in the TCI group was lower (0.845 vs. 1.239), *p* = 0.02 (Figure 2). 

Despite lower remifentanil consumption in the TCI group, this group of patients demonstrated longer total recovery time (Table 3). 

## 4. Discussion

Remifentanil is a very safe drug because of its short time of action and minimal cumulation risk [4]. In our study remifentanil was given by TCI with an approved infusion device incorporating the Minto pharmacokinetic model. However, the Minto model was created for patients with normal body mass index. Therefore, the lower concentration of remifentanil is achieved using Minto model for morbidly obese patients. As such, TCI pump manufacturers will not permit users to input TBW that output BMIs over 42 kg/m^2^ for men, and 35 kg/m^2^ for women. This is an undesired hurdle for anaesthesiologists who manage morbidly obese and extremely obese patients [5]. 

High doses of remifentanil can cause hyperalgesia and increase pain after surgery. These may be the reason why there are few trials of remifentanil use in morbidly obese patients. A group of scientists guided by Tae Kyun Kim saw this tendency and tried to look for the most suitable pharmacokinetic model to use on patients with morbid obesity. They created a new model which included body weight, age and lean body mass. This new method was compared with the Minto and La Colla models and showed more advantages for morbidly obese patients [6].

By comparing the TCI method with MI, we found that a manual infusion of remifentanil produced better results in postoperative patient recovery. Otherwise, higher doses of remifentanil were consumed using manual infusion. In conclusion, the dosage recommended by expert anaesthesiologists is favourable for morbidly obese patients when compared to the TCI method. More studies have to be undertaken in order to evaluate better methods for remifentanil infusion. Otherwise, it seems that not only body mass or lean body mass are important while dosing patients with remifentanil. We think that studies should be conducted in order to discover a new model of calculations, based on patient’s body mass, age and fat body mass. 

To highlight the role of the different remifentanil infusion methods, a larger study should be performed. Our trial included a small amount of patients because of the COVID-19 pandemic and quarantine in Lithuania. Nevertheless, we think that this topic is important and worthy of interest. Further research can improve the quality of service for morbid obesity patients in everyday practice. The appropriate dosing of remifentanil would improve patients’ quality of life and reduce their recovery time after surgery. This leads to better turnover in the operating room. 

## 5. Conclusions

Several anaesthesia-related issues are important in bariatric surgery: deep anaesthesia to provide best conditions for laparoscopic surgery, smooth and predictable recovery with good analgesia and full consciousness to start rehabilitation as early as possible. Nowadays, the success of anaesthesia for morbidly obese patients is based on the anaesthesiologist’s experience. Only a few investigations on dosing morbidly obese patients with remifentanil can be found in the literature. However, more studies have to be conducted and more patients have to be examined in order to improve clinical practice.

## Figures and Tables

**Table 1:** Characteristics of study population.

Variables	MI Group (*n* = 20)	TCI Group (*n* = 11)	*p*-Value
**Age (years)**			
Median (min–max)	40 (28–55)	45 (32–62)	0.203
**BMI (kg/m^2^)**			
Median (min–max)	36.9 (35.003–52.074)	43.8 (38.055–50.926)	0.07
**LBM (kg)**			
Median (min–max)	87.73 (106–71.15)	86.7 (74.89–108.3)	0.596
**Sex**			
Male, *n* (%)	9 (45)	4 (36)	0.654
Female, *n* (%)	11 (55)	7 (64)	

Data are expressed as median ± min/max values, numbers and percentages.

**Table 2:** Remifentanil duration.

Variables	MI Group	TCI Group	*p* Value
Remifentanil duration (min) Median (min–max) Quartiles (Q1–Q3)	73 (50–150) (66–84)	75 (40–128) (78–102)	>0.05

**Table 3:** Intraoperative and immediate postoperative data.

Variables	TCI Group (*n* = 20)	MI Group (*n* = 11)	*p*-Value
**Spontaneous breathing beginning (min)**			
Median (min–max)	8 (4–12)	6 (4–12)	0.317
**Airway reflexes recovery**			
**time (min)**			
Median (min–max)	12 (10–18)	8 (6–16)	0.001
**Extubation time (min)**			
Median (min–max)	12 (10–18)	8 (6–16)	0.001
**Eye opening time (min)**			
Median (min–max)	12 (10–20)	8 (6–16)	0.001
**Recovery of orientation (min)**			
Median (min–max)	14 (12–20)	10 (6–16)	0.001
**Start of the following oral**			
**commands (min)**			
Median (min–max)	14 (12–20)	10 (6–16)	0.001
**Total recovery time (min)**			
Median (min–max)	14 (12–20)	10 (6–16)	0.001

## Data Availability

The data are available on request from the corresponding author. The data are not publicly available due to privacy issues.
